# The 100 Most Cited Kluver-Bucy Research Articles: A Bibliometric Analysis

**DOI:** 10.7759/cureus.45382

**Published:** 2023-09-16

**Authors:** Cynthia Janku, Priya V Engel, Kisan Patel, Elias Giraldo

**Affiliations:** 1 Neurology, California University of Science and Medicine, Colton, USA; 2 Neurosurgery, Arrowhead Regional Medical Center, Colton, USA

**Keywords:** systematic review and meta analysis, bibliometric analyis, medical publications, kluver-bucy, kluver-bucy syndrome

## Abstract

Kluver-Bucy Syndrome (KBS) is a rare neuropsychiatric disorder characterized by hyperorality, hypersexuality, bulimia, visual agnosia, and amnesia due to lesions affecting bilateral temporal lobes. It is attributed to a multitude of causes, including stroke, herpes simplex encephalitis, Alzheimer’s disease, and head trauma. Current treatments for KBS include symptomatic management with antipsychotics, mood stabilizers, carbamazepine, and selective serotonin reuptake inhibitors. The bibliometric analysis was done to reflect the relevance and understanding of KBS in recent literature. The SCOPUS database was utilized to conduct a search for all articles with the terms “Kluver-Bucy” and “Kluver Bucy” from January 1, 1955 (the first available articles from the search) to February 1, 2023. The parameters included in this analysis were article title, citation numbers, citations per year, authors, institutions, publishing journals, country of origin, Source Normalized Impact per Paper, and Scopus CiteScore. Since 1937, when Kluver-Bucy Syndrome was first defined, the publications on KBS have steadily increased, with up to six publications a year in 2002. The most common institutions were SUNY Upstate Medical University, VA Medical Center, and the State University of New York (SUNY) System. Seven of these papers were published in *Neurology*. Almost 75% of the articles were published in journals of medicine and neuroscience. This is the first bibliometric analysis to evaluate the most influential publications about Kluver-Bucy Syndrome. A majority of the research is case-based and there is a dearth of clinical trials to identify the exact pathophysiology and physiotherapy management, possibly owing to the rarity of the disease. Our research suggests that there may be a significant overlap between Sanfilippo syndrome and KBS, suggesting that refined guidelines for establishing diagnosis may be required for children. Our study could bring a renewed interest in this field and lead to additional research focused on understanding the pathophysiology of KBS in order to promote the development of novel diagnostics and treatment.

## Introduction and background

Kluver-Bucy Syndrome (KBS) is a rare neuropsychiatric disorder characterized by hyperorality, hypersexuality, bulimia, visual agnosia, and amnesia due to lesions affecting bilateral temporal lobes [1]. Kluver-Bucy Syndrome has a variety of causes, including stroke, herpes simplex encephalitis, Alzheimer’s disease, and head trauma [2,3]. Current treatments for KBS include symptomatic management with antipsychotics, mood stabilizers, carbamazepine, and selective serotonin reuptake inhibitors (SSRIs) [1]. Research on the pathophysiology, treatment, and management of KBS is sparse, potentially given the rare nature of this disorder. There have been no previous bibliometric analyses on KBS. This analysis was conducted to reflect the relevance and understanding of Kluver-Bucy Syndrome with research trends.

## Review

Methods

The SCOPUS database was utilized to conduct a search for all articles with the terms “Kluver-Bucy” and “Kluver Bucy” from January 1, 1955 (the first available articles from the search) to February 1, 2023. The search was limited to research articles only and excluded reviews, letters, conference papers, editorials, and notes. The parameters included in this analysis were article title, citation numbers, citations per year, authors, first author specialty, institutions, publishing journals, country of origin, Source Normalized Impact per Paper, and Hirsch Index. Self-citations were calculated for each article. Journal impact factors for each year were calculated. To address the association between journal impact factor and the number of total citations, Pearson coefficients were calculated using STATA/BE 17.0, and significance was established as P <0.05 for all calculations.

Results

Systematic Search

**Figure 1:**
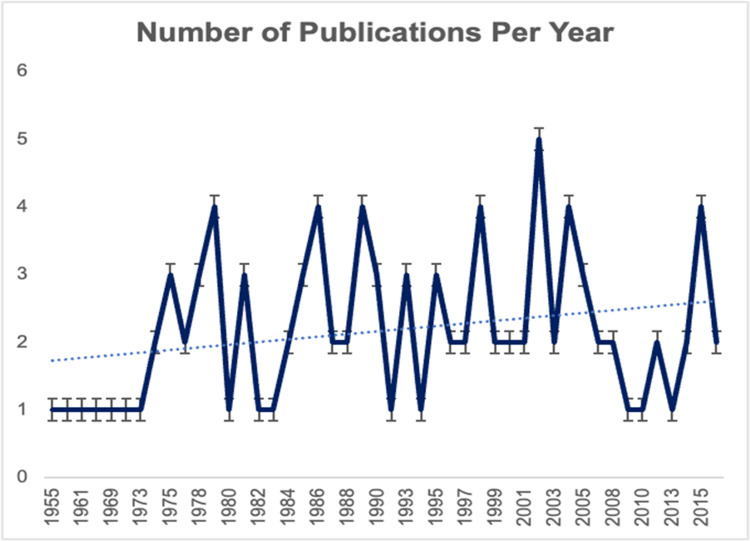
Chart detailing the trends of the number of publications per year from 1955 to 2015

Our search yielded 285 relevant articles. The top 100 are listed below (Table 1). The publication dates for this topic range from 1955 to 2022 (Figure 1). The greatest number of publications occurred between 2000 and 2009 (70 publications, 24.6%). The year with the greatest number of publications was 2001 (12 publications), followed by 2011 (10 publications) and 2014 (9 publications). The average number of citations was 89.1 (standard error (SE) ± 18.0). The range was 1,230, and the median was 36 (interquartile range, 35.5). Analysis of the citations per year yielded a mean of 3.4 and a median of 2.0. The 10 most cited articles were published between 1992 and 2006, ranging from 183 to 1,321 total citations. The most cited article was “Semantic dementia: Progressive fluent aphasia with temporal lobe atrophy,” with 1,578 total citations to date, including 51 self-citations. The article was published in 1992 (Table 1).

**Table 1:** List of the top 100 most-cited Kluver-Bucy Syndrome articles. SJR 2021 = Scientific Journal Rank in 2021

Rank	Title	First Author	Journal title	Year Published	Total Citations	Average Citations per Year	Citations after Removing Self-Citations	Country	SJR 2021
1	Semantic dementia: Progressive fluent aphasia with temporal lobe atrophy	Hodges J.R. et al. [4]	Brain	1992	1578	51	1321	United Kingdom	4.573
2	Visual neurones responsive to faces in the monkey temporal cortex	Perrett D.I. et al. [5]	Experimental Brain Research	1982	957	23	872	United Kingdom	0.621
3	The amygdala theory of autism	Baron-Cohen S. et al. [6]	Neuroscience and Biobehavioral Reviews	2000	818	36	789	United Kingdom	2.66
4	The neuroanatomy of amnesia: A critique of the hippocampal memory hypothesis	Horel J.A. [7]	Brain	1978	357	8	352	United States	4.573
5	Psychiatric phenomena in Alzheimer's disease. IV. Disorders of behaviour	Burns A. et al. [8]	British Journal of Psychiatry	1990	355	11	346	United Kingdom	2.136
6	Distribution of cerebral degeneration in Alzheimer's disease - A clinico-pathological study	Brun A. and Gustafson L. [9]	Archiv für Psychiatrie und Nervenkrankheiten	1976	347	7	311	Sweden	.
7	Working memory for conjunctions relies on the medial temporal lobe	Olson I.R. et al. [10]	Journal of Neuroscience	2006	319	19	309	United States	2.691
8	Syndrome of Klüver and Bucy: Reproduced in man by bilateral removal of the temporal lobes	Terzian H. and Ore G.D. [11]	Neurology	1955	287	4	287	Italy	2.587
9	The human Klüver-Bucy syndrome	Lilly R. et al. [12]	Neurology	1983	241	6	230	United States	2.587
10	Increased social fear and decreased fear of objects in monkeys with neonatal amygdala lesions	Prather M.D. et al. [13]	Neuroscience	2001	217	10	183	United States	1.008
11	Amygdalectomy impairs crossmodal association in monkeys	Murray E.A. and Mishkin M. [14]	Science	1985	183	5	164	United States	14.589
12	Pneumographic findings in the infantile Autism syndrome: A correlation with temporal lobe disease	Hauser S.L. et al. [15]	Brain	1975	180	4	174	United States	4.573
13	Kluver-Bucy syndrome in pick disease: Clinical and pathologic correlations	Cummings J.L. and Duchen L.W. [16]	Neurology	1981	167	4	158	United Kingdom	2.587
14	Acquired Reversible Autistic Syndrome in Acute Encephalopathic Illness in Children	DeLong G.R. et al. [17]	Archives of Neurology	1981	143	3	141	United States	.
15	Complete Klüver-Bucy Syndrome in Man	Marlowe W.B. et al. [18]	Cortex	1975	122	3	122	United States	1.415
16	Semantic memory is an amodal, dynamic system: Evidence from the interaction of naming and object use in semantic dementia	Coccia M. et al. [19]	Cognitive Neuropsychology	2004	103	5	77	Italy	0.919
17	Rett syndrome. Natural history in 70 cases	Naidu S. et al. [20]	American Journal of Medical Genetics	1986	102	3	90	United States	.
18	Partial Klüver-Bucy syndrome produced by destroying temporal neocortex or amygdala	Horel J.A. et al. [21]	Brain Research	1975	99	2	98	United States	0.95
19	Rhinencephalic lesions and behavior in cats. An analysis of the Klüver‐Bucy syndrome with particular reference to normal and abnormal sexual behavior	Green J.D. et al. [22]	Journal of Comparative Neurology	1957	95	1	95	United States	1.31
20	The late effects of necrotizing encephalitis of the temporal lobes and limbic areas: A clinico-pathological study of 10 cases	Hierons R. et al. [23]	Psychological Medicine	1978	87	2	87	United Kingdom, Kiribati	2.328
21	Severe remote memory loss with minimal anterograde amnesia: A clinical note	Stuss D.T. and Guzman D.A. [24]	Brain and Cognition	1988	82	2	81	Canada	0.808
22	The neuropathology of chromosome 17-linked dementia	Sima A.A.F. et al. [25]	Annals of Neurology	1996	80	3	66	United States	3.876
23	Partial Klüver-Bucy syndrome produced by cortical disconnection	Horel J.A. and Keating E.G. [26]	Brain Research	1969	73	1	67	United States	0.95
24	Familial progressive subcortical gliosis	Lanska D.J. et al. [27]	Neurology	1994	67	2	61	United States	2.587
25	Phase II trial of tipifarnib and radiation in children with newly diagnosed diffuse intrinsic pontine gliomas	Haas-Kogan D.A. et al. [28]	Neuro-Oncology	2011	65	5	50	United States	3.097
26	Klüver-bucy syndrome in monkeys with neocortical ablations of temporal lobe	Akert K. et al. [29]	Brain	1961	65	1	65	United States	4.573
27	Kluver-Bucy syndrome after bilateral selective damage of amygdala and its cortical connections	Hayman L.A. et al. [30]	Journal of Neuropsychiatry and Clinical Neurosciences	1998	60	2	60	United States	0.621
28	Familial frontotemporal dementia associated with a novel presenilin-1 mutation	Tang-Wai D. et al. [31]	Dementia and Geriatric Cognitive Disorders	2002	58	3	55	United States	0.855
29	Disorders of facial recognition, social behaviour and affect after combined bilateral amygdalotorny and subcaudate tractotomy- a clinical and experimental study	Jacobson R. [32]	Psychological Medicine	1986	56	2	56	United Kingdom	2.328
30	Limbic dementia	Gascon G.G. and Gilles F. [33]	Journal of Neurology Neurosurgery and Psychiatry	1973	55	1	54	United States	2.992
31	Post-ictal Kluver-Bucy syndrome after temporal lobectomy	Anson J.A. and Kuhlman D.T. [34]	Journal of Neurology Neurosurgery and Psychiatry	1993	54	2	54	United States	2.922
32	The Klüver-Bucy Syndrome in man. A clinico-anatomical contribution to the function of the medial temporal lobe structures.	Pilleri G. [35]	Psychiatria et neurologia	1966	54	1	54	Switzerland	.
33	Neuropathological correlates of behavioural disturbance in confirmed Alzheimer's disease	Forstl H. et al. [36]	British Journal of Psychiatry	1993	53	2	47	Germany	2.136
34	Clinical aspects of argyrophilic grain disease	Ikeda K. et al. [37]	Clinical Neuropathology	2000	52	2	46	Japan	0.313
35	Klüver-Bucy syndrome after left anterior temporal resection	Ghika-Schmid F. et al. [38]	Neuropsychologia	1995	52	2	52	Switzerland	1.14
36	Independent modulation of basal and feeding-evoked dopamine efflux in the nucleus accumbens and medial prefrontal cortex by the central and basolateral amygdalar nuclei in the rat	Ahn S. and Phillips A.G. [39]	Neuroscience	2003	50	3	46	Canada	1.008
37	The amygdala: Functional organization and involvement in neurologic disorders	Benarroch E.E. [40]	Neurology	2015	46	6	45	United States	2.587
38	The association between tick-borne infections, Lyme borreliosis and autism spectrum disorders	Bransfield R.C. et al. [41]	Medical Hypotheses	2008	45	3	36	United States	0.569
39	Leuprolide treatment of sexual aggression in a patient with dementia and the Kluver-Bucy syndrome	Ott B.R. [42]	Clinical Neuropharmacology	1995	44	2	44	United States	0.334
40	Repetitive behaviors in schizophrenia: A single disturbance or discrete symptoms?	Tracy J.I. et al. [43]	Schizophrenia Research	1996	43	2	40	United States	1.451
41	Pleomorphism of the clinical manifestations of neurocysticercosis	Patel R. et al. [44]	Transactions of the Royal Society of Tropical Medicine and Hygiene	2006	42	2	42	England	0.595
42	Postinfectious immune-mediated encephalitis after pediatric herpes simplex encephalitis	De Tiège X. et al. [45]	Brain and Development	2005	42	2	41	Switzerland	0.572
43	Kluver Bucy syndrome in young children	Pradhan S. et al. [46]	Clinical Neurology and Neurosurgery	1998	42	2	42	India	0.571
44	Kluver-Bucy syndrome in Huntington’s chorea	Janati A. [47]	Journal of Nervous and Mental Disease	1985	42	1	42	United States	0.622
45	Reproductive function in temporal lobe epilepsy: The effect of temporal lobectomy	Cogen P.H. et al. [48]	Surgical Neurology	1979	42	1	42	Switzerland	.
46	Comparison of therapeutic effects between selective amygdalohippocampectomy and anterior temporal lobectomy for the treatment of temporal lobe epilepsy: A meta-analysis	Kuang Y. et al. [49]	British Journal of Neurosurgery	2014	41	5	41	England	0.322
47	Kluver-Bucy syndrome - An experience with six cases	Jha S. and Patel R. [50]	Neurology India	2004	41	2	40	India	0.321
48	Infantile autism and the temporal lobe of the brain	Hetzler B.E. and Griffin J.L. [51]	Journal of Autism and Developmental Disorders	1981	41	1	41	Switzerland	1.207
49	The fornix and limbic system	Lövblad K.-O. et al. [52]	Seminars in Ultrasound, CT and MRI	2014	39	4	39	Switzerland	0.38
50	Superior colliculus lesions impair threat responsiveness in infant capuchin monkeys	Maior R.S. et al. [53]	Neuroscience Letters	2011	38	3	28	Brazil, Japan	0.783
51	Regional patterns of cortical blood flow distinguish extraverts from introverts	Stenberg G. et al. [54]	Personality and Individual Differences	1990	38	1	35	Sweden	1.178
52	The Klüver-Bucy syndrome produced by partial isolation of the temporal lobe	Horel J.A. and Misantone L.J. [55]	Experimental Neurology	1974	38	1	35	United States	1.478
53	Hypersexuality after temporal lobe resection	Baird A.D. et al. [56]	Epilepsy and Behavior	2002	37	2	32	Switzerland	0.876
54	Kluver-Bucy syndrome and amyotrophic lateral sclerosis: A case report with biochemistry, morphometrics, and Golgi study	Dickson D.W. et al. [57]	Neurology	1986	37	1	34	United States	2.587
55	Visual discrimination impaired by cutting temporal lobe connections	Horel J.A. and Misantone L.J. [58]	Science	1976	37	1	35	United States	14.589
56	Phase i trial of tipifarnib in children with newly diagnosed intrinsic diffuse brainstem glioma	Haas-Kogan D.A. et al. [59]	Neuro-Oncology	2008	36	2	30	England	3.097
57	The Kluver-Bucy syndrome	Goscinski I. et al. [60]	Journal of Neurosurgical Sciences	1997	36	1	36	Italy	0.625
58	Klüver-Bucy Syndrome: Successful Treatment With Carbamazepine	Hooshmand H. et al. [61]	JAMA: The Journal of the American Medical Association	1974	36	1	36	United States	6.076
59	Kluver-Bucy syndrome, hypersexuality, and the law	Devinsky J. et al. [62]	Neurocase	2010	35	3	33	England	0.324
60	Amygdaloid atrophy in frontotemporal dementia and Alzheimer's disease	Boccardi M. et al. [63]	Neuroscience Letters	2002	35	2	33	Switzerland	0.783
61	Persistent Kluver-Bucy syndrome after bilateral thalamic infarction	Müller A. et al. [64]	Neuropsychiatry, Neuropsychology and Behavioral Neurology	1999	34	1	34	Netherlands	.
62	Neurochemical correlates of the Klüver-Bucy syndrome by in vivo microdialysis in monkey	Kling A.S. et al. [65]	Behavioural Brain Research	1993	34	1	33	United States	6.1
63	Natural history of Kluver-Bucy syndrome after treated herpes encephalitis	Hart R.P. et al. [66]	Southern Medical Journal	1986	34	1	34	United States	0.264
64	The neurobehavioral phenotype in mucopolysaccharidosis Type IIIB: An exploratory study	Shapiro E. et al. [67]	Molecular Genetics and Metabolism Reports	2016	33	5	23	United States	0.575
65	Kluver-Bucy syndrome caused by adreno-leukodystrophy	Powers J.M. et al. [68]	Neurology	1980	33	1	33	United States	1.471
66	Social inference deficits in temporal lobe epilepsy and lobectomy: Risk factors and neural substrates	Cohn M. et al. [69]	Social Cognitive and Affective Neuroscience	2015	32	4	29	England	0.876
67	Excessive masturbation after epilepsy surgery	Ozmen M. et al. [70]	Epilepsy and Behavior	2004	31	2	31	United States	1.186
68	Functional involvement of catecholamines in reward-related neuronal activity of the monkey amygdala	Nakano Y. et al. [71]	Journal of Neurophysiology	1987	31	1	17	United States	1.362
69	Carbamazepine treatment of a patient with Kluver-Bucy syndrome	Stewart J.T. [72]	Journal of Clinical Psychiatry	1985	31	1	30	United States	0.622
70	The Klüver-Bucy syndrome in man	Shraberg D. and Weisberg L. [73]	Journal of Nervous and Mental Disease	1978	31	1	31	United States	.
71	Alzheimer abnormalities of the amygdala with Klüver-Bucy syndrome symptoms: An amygdaloid variant of Alzheimer disease	Kile S.J. et al. [74]	Archives of Neurology	2009	29	2	29	United States	0.95
72	Visual discrimination learning impairments produced by combined transections of the anterior temporal stem, amygdala and fornix in marmoset monkeys	Maclean C.J. et al. [75]	Brain Research	2001	28	1	19	United Kingdom	0.621
73	Selective serotonin reuptake inhibitor treatment of post-traumatic Kluver-Bucy syndrome	Slaughter J. et al. [76]	Brain Injury	1999	28	1	27	United States	1.011
74	Quantifying behaviors of children with Sanfilippo syndrome: The Sanfilippo Behavior Rating Scale	Shapiro E.G. et al. [77]	Molecular Genetics and Metabolism	2015	27	3	18	United States	1.893
75	Hyperorality in epileptic seizures: Periictal incomplete Klüver-Bucy syndrome	Janszky J. et al. [78]	Epilepsia	2005	27	2	25	Hungary	0.334
76	Moclobemide-induced hypersexuality in patients with stroke and Parkinson's disease	Korpelainen J.T. et al. [79]	Clinical Neuropharmacology	1998	27	1	26	Finland	1.697
77	Increased eating in dementia	Hope R.A. et al. [80]	International Journal of Eating Disorders	1989	27	1	23	United Kingdom	0.764
78	Methotrexate leukoencephalopathy presenting as Klüver-Bucy syndrome and uncinate seizures	Antunes N.L. et al. [81]	Pediatric Neurology	2002	26	1	26	United States	0.764
79	Kluver-Bucy syndrome following heat stroke in a 12-year-old girl	Pitt D.C. et al. [82]	Pediatric Neurology	1995	26	1	26	United States	0.187
80	The Phenomenology of the Human Klüver-Bucy Syndrome [Die Phänomenologie des nach Klüver und Bucy benannten Syndroms beim Menschen]	Aichner F. [83]	Fortschritte der Neurologie Psychiatrie	1984	26	1	26	Germany	0.568
81	Mucopolysaccharidosis Type IIIA presents as a variant of Klüver-Bucy syndrome	Potegal M. et al. [84]	Journal of Clinical and Experimental Neuropsychology	2013	24	2	14	United States	0.764
82	Kluver-Bucy syndrome in children	Tonsgard J.H. et al. [85]	Pediatric Neurology	1987	24	1	24	United States	2.922
83	Rejection behaviour: A human homologue of the abnormal behaviour of Denny-Brown and Chambers' monkey with bilateral parietal ablation	Mori E. and Yamadori A. [86]	Journal of Neurology Neurosurgery and Psychiatry	1989	23	1	23	Japan	0.89
84	Visual impairments in macaques following inferior temporal lesions are exacerbated selectively by additional damage to superior temporal sulcus	Aggleton J.P. and Mishkin M. [87]	Behavioural Brain Research	1990	22	1	22	United States	.
85	Obsessive—compulsive disorder and caudate—frontal lesion	Tonkonogy J. and Barreira P. [88]	Neuropsychiatry, Neuropsychology and Behavioral Neurology	1989	22	1	22	United States	0.876
86	Transient Kluver-Bucy syndrome following complex partial status epilepticus	Varon D. et al. [89]	Epilepsy and Behavior	2003	21	1	21	United States	1.324
87	Kluver-Bucy syndrome in a child with bilateral arachnoid cysts: Report of a case	Rossitch E. Jr. and Oakes W.J. [90]	Neurosurgery	1989	21	1	20	United States	0.788
88	Amygdaloid hypersexuality in male cats re-examined	Aronson L.R. and Cooper M.L. [91]	Physiology and Behavior	1979	21	0	21	United States	0.155
89	Further delineation of the KBG syndrome	Devriendt K. et al. [92]	Genetic Counseling	1998	20	1	20	Belgium	0.454
90	The use of superimposed rhythm to decrease the rate of speech in a brain-damaged adolescent	Cohen N.S. [93]	Journal of Music Therapy	1988	20	1	17	United States	.
91	Recovery from a partial Kluver-Bucy syndrome in the monkey produced by disconnection	Horel J.A. and Keating E.G. [94]	Journal of Comparative and Physiological Psychology	1972	20	0	15	United States	0.836
92	Human Kluver-Bucy syndrome following acute subdural haematoma	Yoneoka Y. et al. [95]	Acta Neurochirurgica	2004	18	1	18	Japan	1.362
93	Epileptic Kluver-Bucy syndrome: Case report	Nakada T. et al. [96]	Journal of Clinical Psychiatry	1984	18	0	18	United States	.
94	Acute intermittent porphyria and the Kluver-Bucy syndrome	Guidotti T.L. et al. [97]	Johns Hopkins Medical Journal	1979	18	0	18	United States	1.359
95	Partial Klüver-Bucy syndrome following probable herpes simplex encephalitis	Shoji H. et al. [98]	Journal of Neurology	1979	18	0	18	Japan	0.264
96	Klüver-Bucy syndrome as a result of minor head trauma	Salim A. et al. [99]	Southern Medical Journal	2002	17	1	16	United States	1.4
97	Impaired acquisition of new words after left temporal lobectomy despite normal fast-mapping behavior	Warren D.E. et al. [100]	Neuropsychologia	2016	16	2	14	United States	1.14
98	The neuropsychiatric spectrum of motivational disorders	Epstein J. and Silbersweig D. [101]	Journal of Neuropsychiatry and Clinical Neurosciences	2015	16	2	14	United States	0.621
99	A clinical overview of pseudobulbar affect	Arciniegas D.B. [102]	American Journal Geriatric Pharmacotherapy	2005	16	1	13	United States	.
100	Heinrich Klüver and the temporal lobe syndrome.	Nahm F.K. [103]	Journal of the history of the neurosciences	1997	16	1	1	United States	0.229

Impact Factor

Our analysis indicates that there was a significant association between Scientific Journal Rank (SJR) 2021 and the total number of citations (Table 2). For every 1-point increase in SJR, there were 56.4 additional citations (p<0.01). A similar trend was noted in Scopus’ CiteScore (2022) value which indicated that for every 1-point increase in CiteScore, there were 11.4 additional citations (p<0.05). A similar association was appreciated when analyzing average citation per year. For every 1-point increase in SJR, there were 1.9 more average citations per year (p<0.05) and for every 1-point increase in CiteScore, there were 0.4 more average citations per year (p<0.05).

**Table 2:** Logistic Regression Analyses to determine associations between citations and journal citation rank in the Scientific Journal Rank (SJR) and Cite Score. *Three article anomalies were excluded from this analysis due to a low number of citations in an impactful journal: Klüver-Bucy Syndrome: Successful Treatment With Carbamazepine [61], Amygdalectomy impairs crossmodal association in monkeys [14], Visual discrimination impaired by cutting temporal lobe connections [58].

Variable	Total Citations	95% Confidence Interval	P-Value	Citations without self-citations	95% Confidence Interval	P-Value	Average Citations Per Year	95% Confidence Interval	P-Value
SJR 2021	62.8	26.3-99.4	0.001	56.4	24.2-88.7	0.001	1.9	0.7-3.1	0.003
Cite Score 2022	12.8	4.4-21.2	0.003	11.4	3.9-18.8	0.003	0.4	0.1-0.7	0.006

Journal of Publication

The top 100 cited articles were published in 10 journals (Table 3). The Journal of Neurology published 7% of the articles, followed by Brain (4%). The 2021 SJR for these two journals was 2.6 and 4.6, respectively, while the 2020 Impact factors were 9.9 and 13.5, respectively.

**Table 3:** Table listing the top journals publishing papers on Kluver-Bucy Syndrome.

Journals of Publication	Number of Articles (n=100)
Neurology	7
Brain	4
Brain Research	3
Epilepsy and Behavior	3
Journal of Neurology Neurosurgery and Psychiatry	3
Pediatric Neurology	3
Archives of Neurology	2
Behavioral Brain Research	2
British Journal of Psychiatry	2
Clinical Neuropharmacology	2

Countries and Institutions

**Figure 2:**
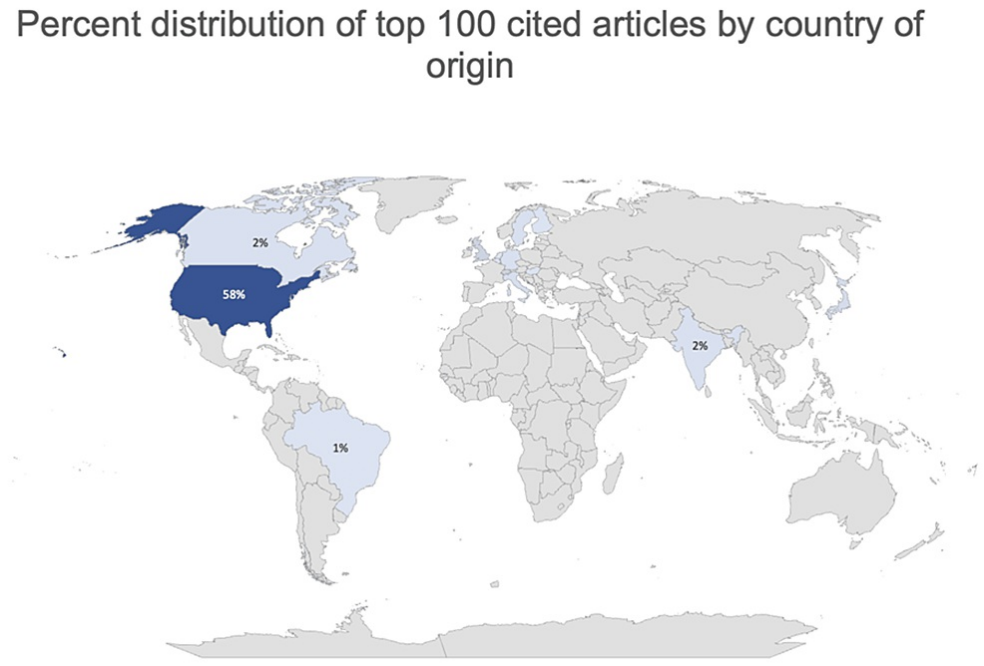
Map detailing the distribution of Top Cited Articles by Country of Origin. The United States has published 58% of the top cited articles. Created on Excel Powermap, Excel, 2023

**Figure 3:**
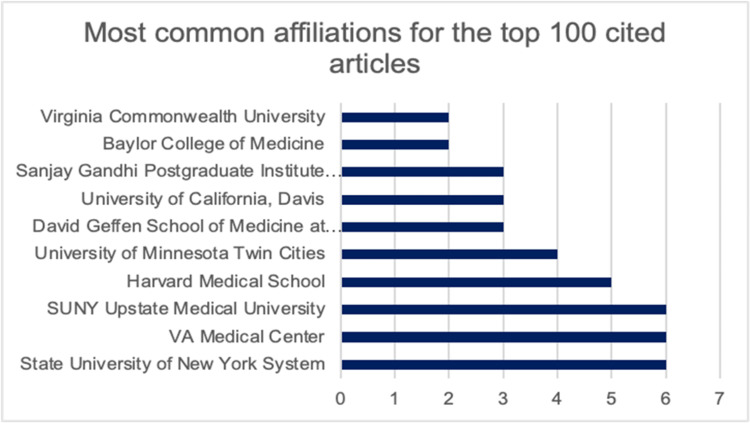
Chart detailing the top affiliations of KBS articles. The most common institutions were SUNY Upstate Medical University, VA Medical Center, and the State University of New York (SUNY) System. KBS: Kluver-Bucy Syndrome

The top 100 cited articles were published by 16 countries. Most of the articles were published by the United States (58.9%), followed by the United Kingdom (8.8%), and Switzerland (7.8%) (Figure 2). There were a total of 160 contributing institutions (Figure 3). Veterans Affairs Medical Center and SUNY Upstate Medical University were the top contributing articles in this analysis, each producing six articles. Harvard Medical School and the University of Minnesota Twin Cities each produced five articles.

Article Subject

**Figure 4:**
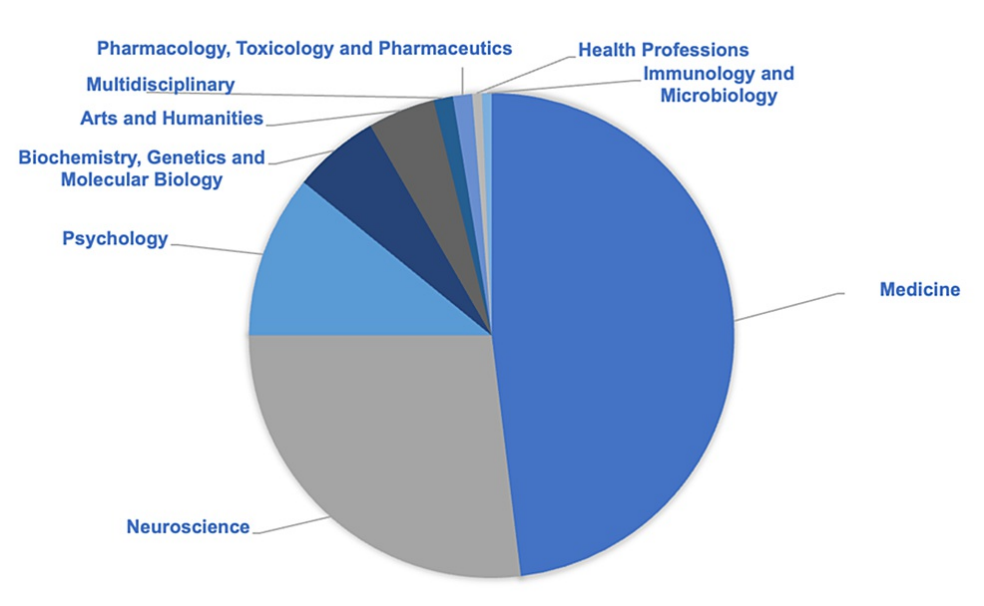
Chart detailing the subject of the articles in the 100 top cited on KBS. Almost 75% of them were in medicine and neuroscience. KBS: Kluver-Bucy Syndrome

SCOPUS’ classification of article subjects yielded that 48.1% of articles were classified as Medicine articles followed by Neuroscience (26.9%) and Psychology (17%) (Figure 4). Other classifications included Biochemistry, Genetics, and Molecular Biology, Arts and Humanities, Multidisciplinary, Pharmacology, Toxicology, and Pharmaceuticals, Health Professions, and Immunology and Microbiology.

Authors

**Figure 5:**
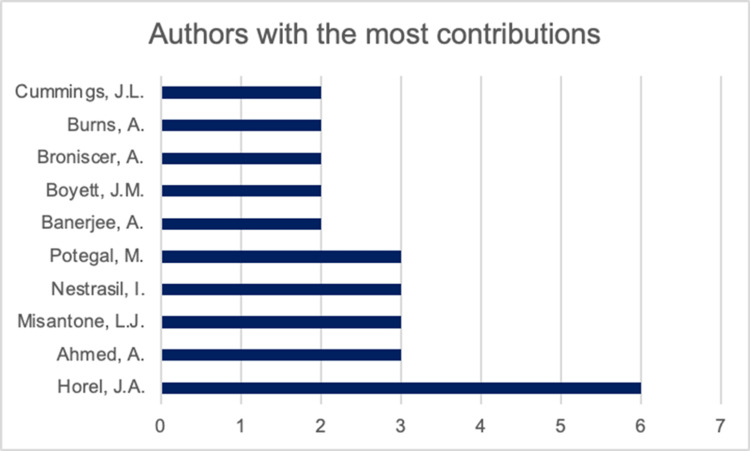
Chart detailing the authors with the most papers published on Kluver-Bucy Syndrome. James A. Horel had the most publications on the subject with six articles. Horel, J. A. [7, 21, 26, 55, 58, 94]; Ahmed, A. [67, 77, 84]; Misantone, L. J. [21, 55, 58]; Potegal, M. [67, 77, 84]; Banerjee, A. [28, 59]; Boyett, J. M. [28, 59]; Broniscer, A. [28, 59]; Burns, A. [8, 36]; Cummings, J. L. [12, 16].

The authors who contributed the most articles in this review were James A Horel (n=6) from SUNY Upstate Medical University, Alia Ahmed (n=3) from the Children’s Hospital in Lahore, Pakistan, Louis J Misantone (n=3) from SUNY Upstate Medical Center, Igor Nestrasil (n=3) and Michael Potegal (n=3) from University of Minnesota Twin Cities. Dr. James A. Horel, PhD had seven publications within the top 100 cited articles with six first authorships and one second authorship. The other listed authors each had three articles in the top 100 (Figure 5).

Discussion

KBS is a rare neuropsychiatric disorder in which destruction of bilateral temporal lobes and amygdala causes hypersexuality, amnesia, visual agnosia, bulimia, hyperorality, hypermetamorphosis, and placidity [1,4]. Partial KBS is defined as having at least three of these symptoms.

Pathophysiology

Multiple papers have described the pathophysiology of KBS, including Alzheimer’s disease, herpes simplex encephalitis, stroke, and head trauma [2,3]. Herpes simplex virus is known to cause dysfunction of the temporal lobes. Costa et al. [2] wrote a case report of a 21-year-old man who developed KBS. This patient presented to the emergency department with seizures. The patient’s MRI revealed bilateral temporal lobe lesions and the patient had positive herpes simplex 1 IgG and IgM antibodies. Once the patient’s neurologic deficits had resolved, he developed the symptoms of KBS. Al-Attas et al. [104] reported a case of a male who developed KBS after a non-dominant middle cerebral artery stroke. The middle cerebral artery feeds the temporal lobes; therefore, the lesion resulted in behaviors consistent with KBS. Alzheimer’s disease is known to cause cortical atrophy. When the amygdala and temporal lobes are affected, KBS can occur [74]. The first reported patient that was described to have KBS had a bilateral temporal lobectomy for the treatment of epileptic seizures [105].

Presentation in Adults vs Children

In 1998, Pradhan et al. [46] described the presentation of KBS in young children who had no previous exposure or educational teachings about sex. The study focused on seven pre-pubertal patients, ages 2.5 to 6 years, who developed KBS as a sequela of herpes simplex encephalitis. The presentations ranged from 10 months to 5.5 years after infection. Incomplete KBS was more likely than complete syndrome, although patients displayed a majority of the symptoms of complete syndrome. All patients displayed alterations in emotions, exemplified by a loss of attachment to family members as well as a range of dispositions from abnormally cheerful to irritable. Dietary changes were also noted with cases of bulimia. Hyperorality was described through a strong propensity to place non-food objects in their mouth. Children displayed hypersexuality in a different way than adults; these manifestations included intermittent pelvic thrusting and fixation on their genitals through frequent holding and rubbing of genitals when in a prone position. Hypermetamorphosis, or easy distractibility through visual stimulation, was found in three of the seven patients. Patients also had abnormal sporadic sharp or spike waves arising from the temporal lobe in sleep electroencephalogram findings. In all patients, the manifestations of the syndrome declined over time [46].

In their 2015 study, Shapiro et al. [77] described Sanfilippo syndrome Type A as a variant of KBS. Sanfilippo syndrome is caused by deficient activity of lysosomal enzymes that result in a degradation of heparan sulfate with symptoms such as progressive dementia, aggressive behavior, hyperactivity, and seizures. Of the four variants of Sanfilippo syndrome, Type A was considered to be the most prevalent. Aggression and a lack of regard for dangerous situations distinguished Type A from other forms of Sanfilippo syndrome that also included cognitive impairment. Measurable volume loss in cortical and subcortical gray matter, particularly in the amygdala, was also seen in these children. The findings suggest that KBS can manifest through different pathophysiological mechanisms and with different presenting symptoms than those previously reported in adults.

Treatment and Management

Current treatments for KBS include symptomatic management with antipsychotics, mood stabilizers, carbamazepine, and SSRIs [1]. Multiple papers have described successful treatment of the symptoms with carbamazepine [61, 72]. Lanska [106] described treatment of KBS as “difficult and unsatisfactory”, given that there is no specific medication that can fully resolve the symptoms of KBS.

Management of Kluver-Bucy syndrome revolves around symptomatic management.

Ott [42] found that the sequelae of temporal lobe lesions may vary in both their severity and presentation, presenting an opportunity for specialized treatment modalities. For example, the use of beta-blocker Propranolol was found to be effective in quelling verbal and physical aggression, while gonadotropin-releasing hormone antagonist Leuprolide effectively controlled hypersexual and inappropriate behaviors. Stewart [72] and Hooshmand et al. [61] highlighted the effectiveness of the anti-seizure medication carbamazepine in symptoms and demonstrated a dramatic reduction in unprovoked episodes of rage. The effect of SSRIs was also investigated and found to have a positive effect on the overall constellation of symptoms [42, 76].

Documented cases vary in the etiologies of temporal lobe lesions, which could play a role in the varied outcomes of various treatment modalities. Larger sample sizes with more standardized treatment protocols are indicated to assess the efficacy of individual treatment options and which symptoms they best target, if not all.

Publication Trends

Since 1937, the publications on KBS have steadily increased, with up to six publications a year in 2002. This study is the first bibliometric providing a detailed analysis on the top 100 most cited articles in the study of KBS. The analysis included the most cited publications, authors, countries, and institutions contributing to the field of KBS research. The analysis also included average citations per year to account for the effects of earlier publications on total number of citations. Currently, there are no NIH grants funding research on KBS. The Genetic and Rare Disease Information Center has minimal information about the disorder [107].

In 1937, Heinrich Klüver and Paul Bucy [108] expanded on experiments from 1881, noting that the removal of temporal lobes in the Macacus rhesus yielded dramatic behavioral changes, including visual agnosia, oral fixation, hypersensitivity to visual stimuli, increased sexual activity, and changes in dietary habits. In 1955, Dr. Hrayr Tersian and Dr. Giuseppe D. Ore [11] first documented similar findings in humans who had a temporal lobectomy. Since that time, research on KBS has steadily increased, with peak research activity between 2000 and 2009 (Figure 1), suggesting that a majority of impactful research on KBS has occurred in the last two decades.

Top Cited Publications

The top cited article, “Semantic Dementia: Progressive fluent aphasia with temporal lobe atrophy” by Hodges et al. [4] was published in Brain in 1992. The paper had 1,578 total citations and 1,321 non-self-citations, with 51 citations per year. The study described the progression of language decline in patients with anterior inferior temporal lobe conditions, discussing KBS as symptoms that developed after suspected Alzheimer’s disease or Pick’s disease. The second most cited article in this analysis was “Visual neurones responsive to faces in the monkey temporal cortex” [5], published in 1982 in Experimental Brain Research. The article had 872 non-self-citations with an average of 23 citations per year. The study discussed that 48 neurons in the superior temporal sulcus were most important in arousal, emotional and motor reactions, which can explain how temporal lobe damage can lead to KBS [5]. The next paper discussed how the “social brain”, including the amygdala, can show that deficits such as KBS can be a model of autism [6].

The bibliometric analysis reflected that most articles came out of the United States, showing that there could be a higher incidence of KBS in the US with more research done on the disorder. VA Medical Centers and SUNY Upstate Medical University published the greatest number of articles, furthering our knowledge of this rare disease.

Limitations

Inherent limitations to bibliometric analyses include limited availability, exclusion of certain formats of publications such as conferences or textbooks, and difficulty in parsing apart authorships. Moreover, newer articles may not have had adequate time to garner enough citations for inclusion in the “top 100” analyses. Focusing our search through Scopus has its limitations of being a singular database to collect the data. KBS is a rare condition, mainly discussed in case reports and series, another limitation of our study.

## Conclusions

In conclusion, we demonstrated the relevance and understanding of KBS in recent literature through our bibliometric analysis of the 100 most cited research articles on KBS. Further research on KBS is warranted, especially with the purpose to find more effective, therapeutic options.
